# Psychometric properties of FACIT-Fatigue in systemic lupus erythematosus: a pooled analysis of three phase 3 randomised, double-blind, parallel-group controlled studies (BLISS-SC, BLISS-52, BLISS-76)

**DOI:** 10.1186/s41687-021-00298-x

**Published:** 2021-04-08

**Authors:** Regina Rendas-Baum, Nishtha Baranwal, Ashish V. Joshi, Josephine Park, Mark Kosinski

**Affiliations:** 1 Optum, Johnston, RI USA; 2 GSK, Value Evidence & Outcomes, Collegeville, PA USA

**Keywords:** FACIT-Fatigue, Fatigue, Patient-reported outcomes, Psychometric, Quality of life, Systemic lupus erythematosus, Validity

## Abstract

**Background:**

Fatigue is a key symptom in patients with systemic lupus erythematosus (SLE), and regulatory bodies recommend its assessment in clinical trials of SLE therapies.

**Methods:**

This post hoc pooled analysis of the three BeLimumab In Subjects with Systemic lupus erythematosus (BLISS) Phase 3 randomised, double-blind, parallel-group controlled trials evaluated the measurement properties of the Functional Assessment of Chronic Illness Therapy (FACIT)-Fatigue. Patients (*N* = 2520) completed the FACIT-Fatigue every 4 weeks from baseline until the end of each study period. Internal consistency, test–retest reliability, convergent validity, and ability to detect changes in SLE were evaluated for the FACIT-Fatigue.

**Results:**

The FACIT-Fatigue showed good internal consistency reliability (Cronbach’s alpha > 0.90), very good test–retest reliability (0.76 ≤ intraclass correlation coefficient ≤ 0.92), and moderate-strong convergent validity (0.49 ≤ |r| ≤ 0.86) against scale and summary measure scores from the Short Form 36 Health Survey Version 2. Correlations between FACIT-Fatigue and British Isles Lupus Assessment Group (BILAG) General/Musculoskeletal scores (0.24 ≤ |r| ≤ 0.43) supported convergent validity. Correlations between FACIT-Fatigue and the Safety of Estrogens in Lupus Erythematosus National Assessment-Systemic Lupus Erythematosus Disease Activity Index (SELENA-SLEDAI) scores and SLE annualised flare rate were weak but in the expected direction (ranging from − 0.02 to − 0.25). Known-groups validity testing showed that the FACIT-Fatigue can significantly discriminate between patient groups with differing scores for SELENA-SLEDAI, BILAG (General and Musculoskeletal) ratings, and Physician’s Global Assessment (PGA). Patients showing improvement in PGA and meeting the BILAG responder criteria had significantly higher mean improvement in FACIT-Fatigue scores than those without improvements in either measure (Week 52 mean score difference [95% confidence interval]: − 4.0 [− 5.0, − 3.0] and −2.2 [−3.1, −1.2], respectively; both *p* < 0.0001). The range of important (i.e. meaningful) change in FACIT-Fatigue, based on multiple anchors, was 3–6 points.

**Conclusions:**

The FACIT-Fatigue demonstrated adequate psychometric properties in patients with SLE. The body of evidence from the three BLISS trials (both pooled and individually) supports the FACIT-Fatigue as a reliable and valid measure of SLE-related fatigue in clinical trials.

**Clinical trial identifiers:**

BLISS-SC (NCT01484496), BLISS-52 (NCT00424476), and BLISS-76 (NCT00410384).

**Supplementary Information:**

The online version contains supplementary material available at 10.1186/s41687-021-00298-x.

## Background

Systemic lupus erythematosus (SLE) is a chronic autoimmune disease characterised by diverse clinical manifestations and flares associated with organ damage [1]. Fatigue is the most common symptom experienced by patients with SLE, affecting approximately 50–86% of the SLE population [2–4]. Fatigue in SLE has a multifactorial aetiology [5], and is considered one of the most debilitating symptoms of the disease by many patients, associated with decreased quality of life and increased work disability [4, 6]. Given the substantial burden of fatigue on patients with SLE, regular assessment is important to ensure it is managed optimally, with strategies tailored to the individual patient [7]. Furthermore, both the US Food and Drug Administration (FDA) and the European Medicines Agency (EMA) recognise that fatigue is an important symptom of SLE, and recommend that this should be assessed in clinical trials of SLE therapies [8, 9], using a reliable patient-reported outcome (PRO) measure [8].

The Functional Assessment of Chronic Illness Therapy (FACIT)-Fatigue questionnaire is a PRO instrument that captures multiple aspects of physical and mental fatigue, and their effects on function and daily living [10]. There is only limited evidence currently available regarding whether the FACIT-Fatigue is an appropriate instrument to measure SLE-related fatigue in clinical trials. Based on this limited evidence, the FACIT-Fatigue appears to have good psychometric properties in patients with SLE [10, 11], and findings from cognitive debriefing interviews demonstrate that items of the FACIT-Fatigue scale are relevant and understood by patients with SLE [10]. In an analysis of data from a 52-weekplacebo-controlled trial in patients with moderately to severely active SLE [12], the FACIT-Fatigue showed good internal consistency reliability (Cronbach’s alpha > 0.95 at all visits) and detected improvement in clinical outcome measures of SLE disease activity (British Isles Lupus Activity Group [BILAG] General and BILAG Musculoskeletal) and worsening in patients’ global assessment [11]. Results also showed significant and longitudinally consistent correlations between the FACIT-Fatigue and other measures including pain intensity and interference, the Short Form 36 Health Survey Version 2 (SF-36v2) Vitality domain, Physical component score and Mental component score, and patients’ global assessment of disease activity, supporting the convergent validity of the FACIT-Fatigue in patients with SLE [11]. Furthermore, a systematic literature review of PRO measures targeting key SLE symptoms and impacts concluded that of the three most relevant fatigue instruments, the FACIT-Fatigue demonstrated the strongest evidence of reliability, validity, and ability to detect change in an SLE population, as well as qualitative evidence of the relevance of item content to patients with SLE [13].

To strengthen the currently available evidence in this area, the present study investigated the psychometric properties (internal consistency, test–retest reliability, convergent validity, known-groups validity, and ability to detect changes in SLE) of the FACIT-Fatigue in SLE using data from three randomised controlled trials (RCTs) of belimumab [14–16], a human immunoglobulin G1-λ monoclonal antibody approved for the treatment of SLE in adults (Europe and USA) and children (USA only) who are receiving standard therapy [17–19].

## Methods

### Study design

This was a post hoc analysis (GSK Study 209013) of data from three Phase 3 randomised, double-blind, parallel-group controlled BeLimumab In Subjects with Systemic lupus erythematosus (BLISS) studies that compared the safety and efficacy of belimumab in patients with SLE. Full details of these three trials have been published elsewhere: BLISS-SC (NCT01484496) [16], BLISS-52 (NCT00424476) [14], and BLISS-76 (NCT00410384) [15].

Briefly, the primary efficacy endpoint in all three trials was the SLE Responder Index (SRI) response rate at Week 52 (this endpoint was met in all trials, and showed a significantly higher SRI response rate with belimumab than placebo) [14–16]. Patients were randomised and treated as follows: 2:1 to subcutaneous belimumab 200 mg (*n* = 556) or placebo (*n* = 280) in BLISS-SC, 1:1:1 to intravenous placebo (*n* = 287) or belimumab 1 mg/kg (*n* = 288) or 10 mg/kg (*n* = 290) (BLISS-52), and 1:1:1 to intravenous placebo (*n* = 275) or belimumab 1 mg/kg (*n* = 271) or 10 mg/kg (*n* = 273) in BLISS-76. All patients also received standard therapy. Patients were considered for inclusion if they were ≥ 18 years of age, had a clinical diagnosis of SLE according to American College of Rheumatology criteria, had active SLE, were autoantibody-positive, and were on a stable SLE treatment regimen (that may have included corticosteroids and/or immunosuppressants). Exclusion criteria included severe lupus nephritis, central nervous system lupus or prior treatment with a B-cell-targeted therapy (including rituximab), intravenous cyclophosphamide, or prednisone. Studies were performed in accordance with the Declaration of Helsinki 2008 and approval of institutional review boards; all patients read and signed an informed consent form in addition to providing verbal consent to participate and be audio recorded during interviews.

### Outcome measures

#### FACIT-Fatigue

The FACIT-Fatigue is a self-administered13-item questionnaire that assesses patient-reported fatigue and its impact upon daily activities and function over the prior 7 days [10]. The questionnaire assesses physical fatigue (e.g. “I feel tired”), functional fatigue (e.g. “trouble finishing things”), emotional fatigue (e.g. “I am frustrated by being too tired to do the things I want to do”), and social consequences of fatigue (e.g. “limits social activity”) [10] (Supplementary Table S1). Patients are asked to answer each of the questions using a 5-pointLikert-type scale (0 = Not at all, 1 = A little bit, 2 = Somewhat, 3 = Quite a bit, and 4 = Very much). Each of the 13 items contributes equally to a single conceptual domain representing fatigue. FACIT-Fatigue total scores are the sum of responses and range from 0 to 52, with lower scores indicating greater fatigue and higher scores indicating less fatigue [10].

In all three studies, patients completed the FACIT-Fatigue every 4 weeks from baseline until the end of each trial period (with the exception of Weeks 56 and 64 for BLISS-76).

The current study used endpoints from the original trials as criterion measures in the evaluation of the FACIT-Fatigue measurement properties. These measures included Safety of Estrogens in Lupus Erythematosus National Assessment-Systemic Lupus Erythematosus Disease Activity Index (SELENA-SLEDAI), a measure of reduction in global disease activity; Physician’s Global Assessment (PGA), which measures overall worsening of the patient’s condition; BILAG, which assesses worsening in specific organ systems; and the SF-36v2, a widely used health-reported quality of life measure, consisting of 8 distinct domains that are subsequently aggregated into two summary scores, representing physical and mental health status. The schedule of assessments for each study is summarised in Supplementary Table S2.

With a few exceptions, the analyses were conducted at baseline (Week 0) and Weeks 24 and 52, as FACIT-Fatigue assessments were performed at these time points across all included studies. Analyses to evaluate ability to detect change used data from baseline through Week 24 and from baseline to Week 52 to calculate BILAG response at Week 24 and Week 52, respectively. Intraclass correlation coefficients (ICCs) were calculated using SELENA-SLEDAI and PGA score data between Weeks 8 and 12.

### Data analysis

The current analysis was a psychometric validation of the FACIT-Fatigue. Data from the intent-to-treat populations of the three trials were used to assess reliability, construct validity and responsiveness (ability to detect change) of the FACIT-Fatigue scale. Post hoc analyses were conducted for each trial separately and for the pooled samples, in accordance with FDA guidance that validation of PRO measures be conducted in samples reflective of the patient populations of the trials in which these measures were used [20]. The similarities in study design of each of the trials, in particular the inclusion/exclusion criteria, and study length enabled pooling of patient-level data, allowing overall estimates to be obtained in a larger sample size than the individual trials. Data analyses were conducted using SAS version 9.4 software (SAS Institute Inc., Cary, NC, USA). Significance testing was two-sided and at a level of 0.05 for all analyses. With the exceptions of the use of BILAG response rate as a criterion measure to evaluate the FACIT-Fatigue’s ability to detect change in SLE and the evaluation of test–retest reliability, statistical analyses were conducted with cross-sectional data from baseline, and Weeks 24 and 52.

#### Internal consistency and test–retest reliability

Cronbach’s alpha and the ICC were calculated with the R/MBESS package (https://www3.nd.edu/~kkelley/site/MBESS.html) and SAS version 9.4 software ‘PROC MIXED’ and used to evaluate internal consistency and test–retest reliability, respectively [21, 22]. Given that the items of the FACIT-Fatigue are answered on a 5-point scale, a polychoric correlation coefficients matrix was used to calculate Cronbach’s alpha [23].

A sample of patients with stable disease activity (based on constant SELENA-SLEDAI and PGA scores between Weeks 8 and 12) was used to calculate the ICC as: $\frac{\upsigma_{\mathrm{s}}^2}{\upsigma_{\mathrm{s}}^2+{\upsigma}_{\mathrm{e}}^2}$ with ${\upsigma}_{\mathrm{s}}^2$ and ${\upsigma}_{\mathrm{e}}^2$, the subject error and measurement errors, respectively, from a random effects model [21].

The minimum standard for acceptable reliability for both internal consistency and test–retest reliability was ≥0.70 [24].

#### Construct validity

Spearman correlations between FACIT-Fatigue scores and the SELENA-SLEDAI scores, annualised flare rate, BILAG General and Musculoskeletal systems ratings, and the SF-36v2 were computed at baseline, and Weeks 24 and 52. Correlations ≥0.30 in absolute value were considered indicative of good convergent validity [25].

Known-groups validity was tested by evaluating differences in mean FACIT-Fatigue scores at baseline, Week 24, and Week 52, across mutually exclusive groups of patients who differed in: (1) SELENA-SLEDAI (< 6, 6–9, ≥10), PGA (none/mild, moderate, severe); (2) BILAG Musculoskeletal and General measure scores (A/B vs C/D/E); (3) normal/high baseline levels of C3 and C4. Analysis of variance was conducted to test for statistically significant (*p* < 0.05) differences in mean FACIT-Fatigue scores across these criterion groups.

#### Confirmatory factor analysis

The measurement model of the FACIT-Fatigue was evaluated further using confirmatory factor analysis (CFA) methods appropriate for categorical data (for more details please see Supplementary Materials).

#### Ability to detect changes in SLE

The ability of the FACIT-Fatigue to detect change was evaluated using two different approaches: (1) by computing correlations between changes in FACIT-Fatigue scores and changes in SF-36v2 scales, SELENA-SLEDAI scores, rate of BILAG response, and PGA, with values interpreted as weak (r < 3.0), moderate (r ≥ 0.3 and < 0.5) or strong (r ≥ 0.5); and (2) by evaluating differences in mean changes in FACIT-Fatigue across change in PGA (improved vs same/worse) and BILAG response rate (≥50% vs < 50% of assessments).

The rate of BILAG responses was evaluated as the ratio of the total number of BILAG responses (i.e. no new BILAG A organ domain score or 2 new BILAG B organ domain scores compared with baseline at the time of assessment) divided by the total number of assessments within the period considered. Estimated mean FACIT-Fatigue change scores and tests of statistical significance for differences between BILAG responder groups or PGA improvement groups at Week 24 and Week 52 were evaluated using the following model:

$$ \Delta  \mathrm{FACIT}-{\mathrm{Fatigue}}_{\mathrm{i}\mathrm{j}}={\upbeta}_0+{\upbeta}_1{\mathrm{GROUP}}_{\mathrm{i}}+{\upbeta}_2{\mathrm{Week}}_{\mathrm{j}}+{\upbeta}_3{\mathrm{GROUP}}_{\mathrm{i}}\bullet {\mathrm{Week}}_{\mathrm{j}}+\mathrm{FACIT}-{\mathrm{Fatigue}}_{\mathrm{Baseline},\mathrm{i}} $$

where ij represents the j^th^ observation for the i^th^ patient, GROUP (either BILAG response rate or PGA improvement) and Week (24 or 52) are fixed effects, and FACIT_Baseline_, _i_ represents a continuous adjustment for the baseline score of i^th^ patient. An unstructured covariance matrix was used to take into account repeated measurements for the same patient [26].

## Results

### Patient population

Data from 836, 865, and 819 patients treated with belimumab or placebo in the BLISS-SC, BLISS-52, and BLISS-76 trials, respectively, were included in this pooled analysis. The demographics and baseline characteristics of patients (*N* = 2520) across the three trials are shown in Table 1. In total, 94% of patients were female, which is in line with the proportion of female participants in the FACIT-Fatigue validation study [11], as well as the general population of patients with SLE [27]. The pooled population had a mean age of 38.1 years and a mean FACIT-Fatigue Total Score of 30.7, and just over half were white. The number of missing FACIT-Fatigue scale scores, and individual items, at each study time point are shown in Supplementary Table S3**.**

**Table 1:** Demographics and baseline characteristics of participants in the three SLE trials

Parameter	BLISS-SC (***n*** = 836)	BLISS-52 (***n*** = 865)	BLISS-76 (***n*** = 819)	Pooled population (N = 2520)
**Age, years, mean (SD)**	38.6 (12.3)	35.5 (11.1)	40.2 (11.5)	38.1 (11.8)
**Female, n (%)**	789 (94.4)	821 (94.9)	764 (93.3)	2374 (94.2)
**Race, n (%)**				
**White**	502 (60.0)	229 (26.5)	569 (69.5)	1300 (51.6)
**Other**	334 (40.0)	636 (73.5)	250 (30.5)	1220 (48.4)
**FACIT-Fatigue Total Score, mean (SD)**	32.0 (11.9)	33.5 (10.2)	26.6 (12.4)	30.7 (11.9)

*FACIT* Functional Assessment of Chronic Illness Therapy, *SD* Standard deviation, *SLE* Systemic lupus erythematosus

### Internal consistency and test–retest reliability

Across the three BLISS trials, estimates for both internal consistency and test–retest reliability exceeded the minimum value for acceptable reliability (0.70). Cronbach’s alphas ranged from 0.94 to 0.96 at baseline, 0.95 to 0.97 at Week 24, and 0.95 to 0.97 at Week 52. Test–retest reliability of FACIT-Fatigue scores was good, with an ICC coefficient of 0.84 in the pooled population and 0.76 (BLISS-52), 0.81 (BLISS-76), and 0.92 (BLISS-SC) in individual trials.

### Construct validity

Across two of the three BLISS trials that used the SF-36, Week 24 FACIT-Fatigue score correlations were strong with SF-36v2 scores (0.60 ≤ |r| ≤ 0.83), of which the largest correlations were observed between the FACIT-Fatigue and the SF-36v2 Vitality scales (Table 2), which is as expected because both instruments measure the underlying construct of energy/fatigue. The correlations between FACIT-Fatigue and the SF-36v2 Physical component summary and Mental component summary measures were very similar in magnitude, suggesting that the FACIT-Fatigue captures both the physical and mental aspects underlying the fatigue experienced by patients with SLE (Table 2).

**Table 2:** Spearman correlations between FACIT-Fatigue and various measures (Week 24)

Domain	BLISS-SC	n	BLISS-52	n	BLISS-76	n	Pooled	n
**SF-36v2**								
Physical component score	N/A	0	0.67	798	0.69	726	0.69	1524
Mental component score	N/A	0	0.74	798	0.70	726	0.70	1524
Physical functioning	N/A	0	0.66	811	0.66	735	0.66	1546
Role physical	N/A	0	0.73	811	0.76	736	0.74	1547
Bodily pain	N/A	0	0.62	813	0.72	734	0.68	1547
General health	N/A	0	0.61	806	0.60	733	0.61	1539
Vitality	N/A	0	0.79	810	0.83	736	0.82	1546
Social functioning	N/A	0	0.72	813	0.76	736	0.74	1549
Role emotional	N/A	0	0.68	810	0.67	733	0.66	1543
Mental health	N/A	0	0.68	810	0.61	736	0.62	1546
**SELENA-SLEDAI**	−0.17	759	− 0.04	809	− 0.16	735	− 0.13	2303
**BILAG General**	0.32	761	0.27	814	0.43	737	0.37	2312
**BILAG Musculoskeletal**	0.41	761	0.24	814	0.30	737	0.34	2312

Correlations: < 0.3, weak; ≤0.3 to < 0.6, moderate-strong; ≥0.6, strong

*BILAG* British Isles Lupus Assessment Group, *FACIT* Functional Assessment of Chronic Illness Therapy, *N/A* Not assessed, *SELENA-SLEDAI* Safety of Estrogens in Lupus Erythematosus National Assessment-Systemic Lupus Erythematosus Disease Activity Index, *SF-36v2* Short Form-36 Health Survey Version 2

At Week 24, correlations between FACIT-Fatigue and SELENA-SLEDAI total scores were weak (ranging from − 0.04 to − 0.17). Correlations between FACIT-Fatigue and BILAG General or BILAG Musculoskeletal scores were low to moderate, ranging from 0.27 to 0.43 and 0.24 to 0.41, respectively (Table 2).

Correlations between mean FACIT-Fatigue score across two time points (baseline and Week 52, and Weeks 24 and 52) and SLE annualised flare rate (|r| ≤ 0.25) were weak, ranging from − 0.02 to − 0.25 (Table 3). In the pooled sample, mean FACIT-Fatigue scores differed across criterion groups derived from SELENA-SLEDAI scores, PGA of SLE severity, and BILAG General and Musculoskeletal ratings, at all three time points (Table 4). Study participants with higher SELENA-SLEDAI scores (indicating more active disease) had lower mean FACIT-Fatigue scores (i.e. felt more fatigued) (Table 4). Mean FACIT-Fatigue scores were higher (indicating less fatigue) among patients who had SLE severity classified as none or mild (according to PGA); in contrast, those classified as having severe SLE by PGA had the lowest mean FACIT-Fatigue score (indicating more fatigue) (Table 4). When assessing both BILAG General and Musculoskeletal ratings, mean FACIT-Fatigue scores were statistically significantly higher (indicating less fatigue) among the less severe group of patients (those with BILAG disease activity grades of C/D/E) (Table 4).

**Table 3:** Spearman correlations between mean FACIT-Fatigue scores, and number of SLE flares by severity and trial

Trial period	Flare severity	BLISS-SC	BLISS-52	BLISS-76
		***n*** **= 763**	***n*** **= 886**	***n*** **= 828**
**Number of FACIT-Fatigue assessments**		5341	6202	8280
**Weeks 0–52**	All flares	−0.09	−0.14	−0.20
	Mild/moderate	−0.09	− 0.11	− 0.19
	Severe	−0.02	− 0.10	− 0.06
		**n = 763**	***n*** **= 818**	***n*** **= 753**
**Number of FACIT-Fatigue assessments**		2289	2454	3765
**Weeks 24–52**	All flares	−0.14	−0.16	−0.25
	Mild/moderate	−0.14	−0.16	− 0.25
	Severe	−0.04	−0.05	− 0.06

Correlations: < 0.3, weak; ≤0.3 to < 0.6, moderate-strong; ≥0.6, strong

The FACIT-Fatigue scores are patient level mean scores at two time points, mean of 0- and 52-week scores and mean of 24- and 52-week scores

*FACIT* Functional Assessment of Chronic Illness Therapy, *SLE* systemic lupus erythematosus

**Table 4:** Mean FACIT-Fatigue scores across various criterion measures (pooled population)

Week	Patients, n	Category	Mean [95% CI]	***F***-value	***p-***value^**a**^
	**SELENA-SLEDAI**				
**0**	2489	**< 6**	33.8 [31.8, 35.9]	6.0	0.0025*
		**6–9**	31.0 [30.3, 31.8]		
		**≥10**	30.2 [29.6, 30.8]		
**24**	2313	**< 6**	37.2 [36.6, 37.9]	23.8	< 0.0001**
		**6–9**	34.7 [33.9, 35.5]		
		**≥10**	33.1 [32.0, 34.2]		
**52**	1990	**< 6**	37.6 [37.0, 38.3]	33.2	< 0.0001**
		**6–9**	34.4 [33.6, 35.3]		
		**≥10**	32.0 [30.5, 33.4]		
	**PGA**				
**0**	2489	**None/mild**	33.8 [32.5, 35.2]	24.1	< 0.0001**
		**Moderate**	31.0 [30.4, 31.5]		
		**Severe**	27.8 [26.6, 28.9]		
**24**	2313	**None/mild**	38.1 [37.5, 38.8]	78.2	< 0.0001**
		**Moderate**	33.4 [32.8, 34.1]		
		**Severe**	26.4 [23.9, 28.8]		
**52**	1990	**None/mild**	37.9 [37.3, 38.5]	64.8	< 0.0001**
		**Moderate**	32.8 [32.0, 33.7]		
		**Severe**	26.3 [23.2, 29.4]		
	**BILAG General A/B**				
**0**	2489	**No**	31.6 [31.1, 32.1]	132.8	< 0.0001
		**Yes**	22.7 [21.3, 24.1]		
**24**	2313	**No**	36.0 [35.6, 36.5]	90.6	< 0.0001
		**Yes**	24.3 [22.0, 26.7]		
**52**	1990	**No**	36.2 [35.7, 36.7]	63.0	< 0.0001
		**Yes**	23.6 [20.6, 26.7]		
	**BILAG Musculoskeletal A/B**				
**0**	2489	**No**	34.0 [33.2, 34.8]	108.2	< 0.0001
		**Yes**	28.9 [28.4, 29.5]		
**24**	2313	**No**	37.2 [36.7, 37.8]	175.5	< 0.0001
		**Yes**	30.0 [29.0, 30.9]		
**52**	1990	**No**	37.5 [36.9, 38.0]	162.8	< 0.0001
		**Yes**	29.7 [28.6, 30.7]		

^a^*p-*value testing: H0: μi = μj, for all i,j

*p < 0.05 for < 6 versus 6–9 (2.9; 95% CI: [0.7, 5.2]) or ≥ 10 (3.8; 95% CI: [1.6, 5.9]); **p < 0.05 for all pairwise comparisons

Spearman rank-order correlations were used to assess the relationship between changes in FACIT-Fatigue and the SELENA-SLEDAI and PGA

*BILAG* British Isles Lupus Assessment Group, *CI* confidence interval, *FACIT* Functional Assessment of Chronic Illness Therapy, *PGA* Physician’s Global Assessment, *SELENA-SLEDAI* Safety of Estrogens in Lupus Erythematosus National Assessment-Systemic Lupus Erythematosus Disease Activity Index

In the CFA, item-to-factor loadings were acceptable, with only 5 of a total of 78 loadings (13 items evaluated at two time points across the three trials) lower than 0.6, and fit statistics generally supporting a unidimensional model for the FACIT-Fatigue items (details of the CFA are provided in the Supplementary Tables S4 and S5).

### Ability to detect changes in SLE

At Week 52, correlations between the change in FACIT-Fatigue scores and the change in SF-36v2 scale scores were moderate to strong and in the expected direction (ranging from 0.42 to 0.67), with the strongest correlation seen between the FACIT-Fatigue and Vitality subscale (Table 5). The correlations observed between changes in FACIT-Fatigue scores and changes in the clinical assessments of SLE were considerably smaller than those observed with the SF-36v2 scales, ranging from − 0.08 to − 0.12 (SELENA-SLEDAI) and − 0.12 to − 0.31 (PGA) (Table 5). Findings were similar for Week 24 assessments (data not shown).

**Table 5:** Spearman correlations between change from baseline in FACIT-Fatigue and various measures (Week 52)

PRO scale	BLISS-SC (***n*** = 654–662)	BLISS-52 (***n*** = 665–688)	BLISS-76 (***n*** = 606–613)	Pooled (***N*** = 1271–1958)
**SF-36v2**				
Physical component score	N/A	0.48	0.48	0.48
Mental component score	N/A	0.54	0.54	0.53
Physical functioning	N/A	0.42	0.45	0.43
Role physical	N/A	0.46	0.54	0.50
Bodily pain	N/A	0.49	0.50	0.50
General health	N/A	0.46	0.45	0.45
Vitality	N/A	0.63	0.70	0.67
Social functioning	N/A	0.46	0.54	0.50
Role emotional	N/A	0.43	0.43	0.42
Mental health	N/A	0.48	0.48	0.48
**SELENA-SLEDAI**	−0.12	−0.10	−0.08	−0.10
**PGA**	−0.12	−0.17	−0.31	−0.20

Spearman correlations were assessed as weak (r < 3.0), moderate (r ≥ 0.3 and < 0.5) or strong (r ≥ 0.5)

*FACIT* Functional Assessment of Chronic Illness Therapy, *N/A* Not assessed, *PGA* Physician’s Global Assessment, *PRO* Patient-reported outcomes, *SELENA-SLEDAI* Safety of Estrogens in Lupus Erythematosus National Assessment-Systemic Lupus Erythematosus Disease Activity Index, *SF-36v2* Short Form-36 Health Survey Version 2

Across the three trials, improvements in FACIT-Fatigue scores at Week 24 and Week 52 were significantly greater (*p* < 0.0001) among patients with, compared with those without, an improvement in PGA (Table 6). At both Weeks 24 and 52, the difference in mean change from baseline in FACIT-Fatigue scores between these PGA groups was at the threshold of meaningful change of 3–4 points established for the FACIT-Fatigue [11].

**Table 6:** Mean change from baseline in FACIT-Fatigue scores by improvement in PGA and BILAG response rate (pooled sample)

Week		Mean change	[95% CI]	Δ^**a**^	95% CI	***p-***value
**PGA improvement**						
**Week 24**	**No** **(*n*** **= 972)**	2.1	[1.4, 2.7]	−3.9	[−4.7, −3.0]	< 0.0001
	**Yes** **(*n*** **= 1496)**	5.9	[5.4, 6.4]			
**Week 52**	**No** **(*n*** **= 861)**	1.9	[1.1, 2.8]	−4.0	[−5.0, −3.0]	< 0.0001
	**Yes** **(*n*** **= 1481)**	5.9	[5.4, 6.4]			
**BILAG response ≥ 50%**^**b**^						
**Week 24**	**No** **(*n*** **= 835)**	2.7	[2.0, 3.4]	−2.7	[−3.6, −1.9]	< 0.0001
	**Yes** **(*n*** **= 1611)**	5.4	[4.9, 5.9]			
**Week 52**	**No** **(*n*** **= 838)**	3.3	[2.5, 4.1]	−2.2	[−3.1, −1.2]	< 0.0001
	**Yes** **(*n*** **= 1528)**	5.5	[4.9, 6.0]			

^a^Δ (difference) calculated as (improvement in PGA – no improvement in PGA) or (BILAG responder < 50% – BILAG responder ≥50%). Analyses to evaluate ability to detect change used data from baseline through Week 24 and from baseline to Week 52 to calculate BILAG response at Week 24 and Week 52, respectively

^b^Based on a total of 7 and 14 assessments per patient between baseline and Weeks 24 and 52, respectively

*BILAG* British Isles Lupus Assessment Group, *CI* confidence interval, *FACIT* Functional Assessment of Chronic Illness Therapy, *PGA* Physician’s Global Assessment, *W* Week

Study participants who had a BILAG response in at least 50% of assessments between baseline and Week 24 or baseline and Week 52 had significantly greater mean improvements in FACIT-Fatigue scores compared with those who had a BILAG response less frequently (Table 6). Mean [95% confidence interval] improvement in FACIT-Fatigue scores for BILAG responders (5.4 [4.9, 5.9] for baseline–Week 24 and 5.5 [4.9, 6.0] for baseline–Week 52) exceeded the meaningful threshold of change established for the FACIT-Fatigue [11].

## Discussion

Fatigue remains an important concern for patients with SLE, even those who have mild disease, and is considered one of the most debilitating symptoms of the disease by many patients, associated with decreased quality of life and increased work disability [4, 6]. Fatigue in people with SLE is a complex issue and has a multifactorial aetiology, being associated with physical activity, obesity, sleep quality, depression, anxiety, and cognitive dysfunction or can be related to the treatments for SLE itself [5]. Consequently, there is a need for a reliable PRO measure to clearly describe fatigue in SLE including physical and mental components, and measures of fatigue symptoms and effects in the presence of comorbid factors (e.g. depression and medication) [8]. The results of the present post hoc psychometric analysis from the three BLISS RCTs of belimumab build on the limited evidence from clinical trials and qualitative research studies of patients with SLE, showing that the FACIT-Fatigue scale is capable of capturing the multi-dimensional nature of SLE-related fatigue and includes assessment of the physical and mental symptoms of fatigue and their impact on the patient’s daily life. It is true that the results of psychometric analyses alone do not indicate which parameter a particular instrument measures. This aspect can be informed by the evidence from previous qualitative analyses, which were overall supportive of the content validity of the FACIT-Fatigue, even if patients with SLE raised some questions about 4 of the 13 items (“feeling listless”, “having energy”, “too tired to eat”, “needing help doing one’s usual activities”) [10].

In the present analysis, the FACIT-Fatigue demonstrated good internal consistency reliability and very good test–retest reliability in SLE. Convergent validity was supported by strong correlations between the FACIT-Fatigue and SF-36v2 scale and summary measure scores, as well as moderate correlations with BILAG General and Musculoskeletal system scores. Tests of known-groups validity showed that the FACIT-Fatigue can discriminate between groups of study participants who differed in categories of SELENA-SLEDAI scores, BILAG (General and Musculoskeletal) ratings, and PGA. These results strengthen the validity of the FACIT-Fatigue scale to reliably and reproducibly assess fatigue among patients with SLE and its ability to detect treatment-related changes in these patients. Results from the pooled BLISS analysis were similar to those observed in each individual BLISS clinical trial, which highlights the robustness of the findings.

Calculation of Cronbach’s alpha based on the polychoric correlation matrix may overestimate reliability, because it does not take into account possible inaccuracies due to the ordinal nature of the items. It is based on the underlying variable that is assumed to be normally distributed, and not the observed variable, which is used to calculate the FACIT-Fatigue score [28], a concept that is widely used in various statistical models, as the ordinal model using the logit link [29]. An advantage of using Cronbach’s alpha based on the Spearman correlation coefficient is that it can be seen as a lower bound of the reliability coefficient [30]. Our results, which included estimates of reliability that used both types of correlation coefficients, consistently demonstrate very good reliability coefficient estimates, supporting the use of the FACIT-Fatigue score in assisting group-baseddecision-making.

The FACIT-Fatigue has also been used in populations of patients with other autoimmune diseases, including primary Sjögren’s Syndrome [31–33], psoriatic arthritis [34], and rheumatoid arthritis (RA) [35]. In these studies, correlations between the FACIT-Fatigue and other measures of fatigue [31], or the SF-36 [31, 33], were at least moderate (|r| ≥0.30).

Given the substantial burden of fatigue in patients with SLE, it is important to determine whether therapies to treat the condition result in improvements in fatigue that are meaningful to patients. Consequently, it is important to ascertain the minimal clinically important difference (MCID) for the FACIT-Fatigue score, or the smallest change in this score that represents a change in symptoms that is clinically meaningful to the patient. The present analysis demonstrated FACIT-Fatigue score changes of 5.9 points at both Weeks 24 and 52, respectively, in patients with PGA improvements. For patients with BILAG response in ≥50% of assessments, mean change in FACIT-Fatigue was equal to 5.4 and 5.5 points at Weeks 24 and 52, respectively. These values are within the range of prior minimal important difference estimates of 3–6 points, specifically patients with SLE, derived by Lai et al. (2011) [11]. It should be noted that in that study, the authors determined that the MCID was 3–4 points, based on previously published minimal important difference estimates, derived in patients with cancer or RA. Any variations in anchor-based MCID estimates likely reflect the choice of anchors selected for estimation [11]; therefore, based on the available evidence from prior studies and the present one, we would consider the 3–4 points estimate to be the correct MCID in patients with SLE. Future studies using estimation of the MCID based on different anchors may be of interest to further clarify the strength of this finding. The present analysis has some limitations that should be considered. While modern psychometric methods, such as item-response theory, can provide additional insights and improved understanding of how each item of the FACIT-Fatigue contributes to the measurement of the underlying latent variable, our evaluation was limited to classical test theory methods. The generalisability of the findings to the general lupus population may be limited because the findings were based on analysis of data from patients participating in the BLISS RCTs. These studies excluded patients with severe lupus nephritis and central nervous system lupus; as such, these high-risk patients are not represented in the present analysis. Despite these caveats, the results we obtained converged across the three trials suggesting that the findings withstand minor differences across samples. Further assessment of results by study did not in any way contradict or fundamentally change the results based on pooled data.

## Conclusion

This post hoc analysis of the BLISS trials demonstrated measurement properties of the FACIT-Fatigue that are adequate for the assessment of fatigue in patients with SLE, consistently showing degrees of reliability that make it appropriate for use in decision-making about fatigue at the group level, such as in clinical trials. The FACIT-Fatigue was mildly related to clinical outcome measures of disease activity and moderately correlated to all health-related quality of life domains and global health assessments, without clear differentiation. Overall, this comprehensive analysis consolidates the limited existing body of evidence supporting the use of the FACIT-Fatigue to measure fatigue in patients with SLE in clinical trials.

## Supplementary Information

**Additional file 1: Supplementary Table S1.** FACIT-Fatigue scale 13-item questionnaire [10].

**Additional file 2: Supplementary Table S2.** Schedule of assessments for the individual study populations.

**Additional file 3: Supplementary Table S3.** Number of missing FACIT-Fatigue scale scores at each visit by study and for the pooled analysis population (A), and items at each study time point and overall (B).

**Additional file 4:** The confirmatory factor analysis method is described in the text before Supplementary Table S3. Consider adjusting the order and naming of the supplementary files.

**Additional file 5: Supplementary Table S4.** CFA tests of one-factor model, FACIT-Fatigue.

**Additional file 6: Supplementary Table S5.** Model fit statistic for CFA tests of the two-factor model, FACIT-Fatigue.

## Data Availability

The datasets generated and/or analysed during the current study are not publicly available because GSK do not host data in their clinical trial repository for any study that does not assess a GSK medicine (i.e. a TIER 1 study). This post hoc analysis (GSK study 209013) did not evaluate a drug, but rather evaluated the measurement properties of a questionnaire used in three clinical trials (BLISS-SC, BLISS-52 and BLISS-76), and was therefore not registered in Clinicaltrials.gov (note that the original clinical trials were registered: BLISS-SC [NCT01484496], BLISS-52 (NCT00424476) and BLISS-76 [NCT00410384]). However, these data are available from the corresponding author upon reasonable request.

## Abbreviations

*BILAG*: British Isles Lupus Assessment Group
*CFA*: Confirmatory Factor Analysis
*CI*: confidence interval
*EMA*: European Medicines Agency
*FACIT*: Functional Assessment of Chronic Illness Therapy
*FDA*: US Food and Drug Administration
*ICC*: Intraclass correlation coefficient
*MCID*: Minimal clinically important difference
*PGA*: Physician’s Global Assessment
*PRO*: Patient-reported outcome
*RCT*: Randomised controlled trial
*SELENA-SLEDAI*: Safety of Estrogens in Lupus Erythematosus National Assessment-Systemic Lupus Erythematosus Disease Activity Index
*SF-36v2*: Short Form-36 Health Survey Version 2
*SLE*: Systemic lupus erythematosus
*SRI*: SLE Responder Index
