# Supplementary material for: Psychometric properties of FACIT-Fatigue in systemic lupus erythematosus: a pooled analysis of three phase 3 randomised, double-blind, parallel-group controlled studies (BLISS-SC, BLISS-52, BLISS-76)
Source: J Patient Rep Outcomes. 2021 Apr 8;5:33. doi: 10.1186/s41687-021-00298-x (PMC8032841; doi:10.1186/s41687-021-00298-x)
Supplement: Supplementary file 1 — Additional file 1: Supplementary Table S1. FACIT-Fatigue scale 13-item questionnaire [10]. [file 41687_2021_298_MOESM1_ESM.docx]

**Supplementary Table S1.** FACIT-Fatigue scale 13-item questionnaire

| 1. I feel fatigued |
| --- |
| 2. I feel weak all over |
| 3. I feel listless (“washed out”) |
| 4. I feel tired |
| 5. I have trouble starting things because I am tired |
| 6. I have trouble finishing things because I am tired |
| 7. I have energy |
| 8. I am able to do my usual activities |
| 9. I need to sleep during the day |
| 10. I am too tired to eat |
| 11. I need help doing my usual activities |
| 12. I am frustrated by being too tired to do the things I want to do |
| 13. I have to limit my social activity because I am too tired |

# *FACIT* Functional Assessment of Chronic Illness Therapy.

Table reproduced with permission of FACIT.org. A full copyright statement for the FACIT-Fatigue scale 13-item questionnaire is included below. Further information is available at [www.facit.org](http://www.facit.org).

**Copyright statement for FACIT-Fatigue scale 13-item questionnaire**

All translations, adaptations, symptom indices, computer programs, and scoring algorithms, and any other related documents of the FACIT Measurement System, including the Functional Assessment of Cancer Therapy (FACT), are owned and copyrighted by, and the intellectual property of, David Cella, Ph.D. Copyright protection is also extended to electronic versions of all FACIT documents and products.

No changes to the wording or phrasing of any FACIT document can occur without written permission. If any changes are made to the wording or phrasing of any FACIT item without permission, the document cannot be considered the FACIT, and subsequent analyses and/or comparisons to other FACIT data will not be considered appropriate.

Permission to use the name “FACIT” will not be granted for any unauthorized translations of the FACIT or FACIT items. Any analyses or publications of unauthorized changes or translated versions may not use the FACIT name. Any unauthorized translation will be considered a violation of copyright protection.

The FACIT copyright information provided on these documents must be included on every page of a FACIT questionnaire in study documents, and in any reproductions for manuscript or other publication purposes.

If there are issues of scientific or copyright misconduct in using the FACIT system of questionnaires, Dr. Cella reserves the right to withdraw permission for use and seek damages to the full extent provided by international copyright law.

Translation and linguistic validation of all FACIT scales must be performed by FACITtrans.
