# Supplementary material for: Psychometric properties of FACIT-Fatigue in systemic lupus erythematosus: a pooled analysis of three phase 3 randomised, double-blind, parallel-group controlled studies (BLISS-SC, BLISS-52, BLISS-76)
Source: J Patient Rep Outcomes. 2021 Apr 8;5:33. doi: 10.1186/s41687-021-00298-x (PMC8032841; doi:10.1186/s41687-021-00298-x)
Supplement: Supplementary file 2 — Additional file 2: Supplementary Table S2. Schedule of assessments for the individual study populations. [file 41687_2021_298_MOESM2_ESM.docx]

**Supplementary Table S2.** Schedule of assessments for the individual study populations

**BLISS-SC**

|  | **Week** | | | | | | | | | | | | | |
| --- | --- | --- | --- | --- | --- | --- | --- | --- | --- | --- | --- | --- | --- | --- |
|  | **0** | **4** | **8** | **12** | **16** | **20** | **24** | **28** | **32** | **36** | **40** | **44** | **48** | **52** |
| **SELENA-SLEDAI** | x | x | x | x | x | x | x | x | x | x | x | x | x | x |
| **PGA** | x | x | x | x | x | x | x | x | x | x | x | x | x | x |
| **BILAG** | x | x | x | x | x | x | x | x | x | x | x | x | x | x |
| **SLE Flare Index** | x | x | x | x | x | x | x | x | x | x | x | x | x | x |
| **FACIT-Fatigue Scale** | x | x | x | x |  |  | x |  |  | x |  |  |  | x |
| **C-SSRS** | x | x | x | x | x | x | x | x | x | x | x | x | x | x |

**BLISS-52**

|  | **Week** | | | | | | | | | | | | | |
| --- | --- | --- | --- | --- | --- | --- | --- | --- | --- | --- | --- | --- | --- | --- |
|  | **0** | **4** | **8** | **12** | **16** | **20** | **24** | **28** | **32** | **36** | **40** | **44** | **48** | **52** |
| **SELENA-SLEDAI** | x | x | x | x | x | x | x | x | x | x | x | x | x | x |
| **PGA** | x | x | x | x | x | x | x | x | x | x | x | x | x | x |
| **BILAG** | x | x | x | x | x | x | x | x | x | x | x | x | x | x |
| **SLE Flare Index** | x | x | x | x | x | x | x | x | x | x | x | x | x | x |
| **SF-36** | x | x | x | x |  |  | x |  |  | x |  |  |  | x |
| **FACIT-Fatigue Scale** | x | x | x | x |  |  | x |  |  | x |  |  |  | x |
| **EQ-5D** | x | x | x | x |  |  | x |  |  | x |  |  |  | x |
| **Workplace Productivity Questionnaire** | x | x | x | x | x | x | x | x | x | x | x | x | x | x |

**BLISS-76**

|  | **Week** | | | | | | | | | | | | | | | | | | | |
| --- | --- | --- | --- | --- | --- | --- | --- | --- | --- | --- | --- | --- | --- | --- | --- | --- | --- | --- | --- | --- |
|  | **0** | **4** | **8** | **12** | **16** | **20** | **24** | **28** | **32** | **36** | **40** | **44** | **48** | **52** | **56** | **60** | **64** | **68** | **72** | **76** |
| **SELENA-SLEDAI** | x | x | x | x | x | x | x | x | x | x | x | x | x | x |  | x |  | x | x | x |
| **PGA** | x | x | x | x | x | x | x | x | x | x | x | x | x | x |  | x |  | x | x | x |
| **BILAG** | x | x | x | x | x | x | x | x | x | x | x | x | x | x |  | x |  | x | x | x |
| **SLE Flare Index** | x | x | x | x | x | x | x | x | x | x | x | x | x | x |  | x |  | x | x | x |
| **SF-36** | x | x | x | x |  | x | x |  | x |  | x |  | x | x |  |  |  | x |  | x |
| **FACIT-Fatigue Scale** | x | x | x | x |  | x | x |  | x |  | x |  | x | x |  |  |  | x |  | x |
| **EQ-5D** | x | x | x | x |  | x | x |  | x |  | x |  | x | x |  |  |  | x |  | x |
| **Workplace Productivity Questionnaire** | x | x | x | x | x | x | x | x | x | x | x | x | x | x | x | x | x | x | x | x |

*BILAG* British Isles Lupus Assessment Group index, *C-SSRS* Columbia-Suicide Severity Rating Scale, *EQ-5D* EuroQol-5D, *FACIT* Functional Assessment of Chronic Illness Therapy, *PGA* Physician’s Global Assessment, *SELENA-SLEDAI* Safety of Estrogens in in Lupus Erythematosus National Assessment-Systemic Lupus Erythematosus Disease Activity Index, *SF-36* Short Form-36 Health Survey, *SLE* Systemic Lupus Erythematosus.

The FACIT-Fatigue was administered across all three trials at baseline and Weeks 4, 8, 12, 24, and 52.
