# Supplementary material for: Psychometric properties of FACIT-Fatigue in systemic lupus erythematosus: a pooled analysis of three phase 3 randomised, double-blind, parallel-group controlled studies (BLISS-SC, BLISS-52, BLISS-76)
Source: J Patient Rep Outcomes. 2021 Apr 8;5:33. doi: 10.1186/s41687-021-00298-x (PMC8032841; doi:10.1186/s41687-021-00298-x)
Supplement: Supplementary file 3 — Additional file 3: Supplementary Table S3. Number of missing FACIT-Fatigue scale scores at each visit by study and for the pooled analysis population (A), and items at each study time point and overall (B). [file 41687_2021_298_MOESM3_ESM.docx]

**Supplementary Table S3.** Number of missing FACIT-Fatigue scale scores at each visit by study and for the pooled analysis population (A), and items at each study time point and overall (B)

**A**

| **Study** | **Week** | **Total responses, N** | **Missing responses, n (%)** |
| --- | --- | --- | --- |
| **BLISS-SC** | **0** | 834 | 2 (0.2) |
|  | **24** | 762 | 1 (0.1) |
|  | **52** | 680 | 2 (0.3) |
| **BLISS-52** | **0** | 843 | 22 (2.6) |
|  | **24** | 814 | 71 (8.7) |
|  | **52** | 691 | 174 (25.2) |
| **BLISS-76** | **0** | 812 | 7 (0.9) |
|  | **24** | 737 | 91 (12.3) |
|  | **52** | 619 | 201 (32.5) |
| **Pooled analysis population** | **0** | 2489 | 31 (1.2) |
|  | **24** | 2313 | 163 (7.0) |
|  | **52** | 1990 | 377 (18.9) |

**B**

|  |  | **Total responses/missing items by study time point** | | | | | | | |
| --- | --- | --- | --- | --- | --- | --- | --- | --- | --- |
|  |  | **Week 0 N/n** | | **Week 24  N/n** | | **Week 52  N/n** | | **All  N/n** | |
| **BLISS-SC** | 3. I feel listless (“washed out”) | 833 | 3 | 762 | 1 | 680 | 2 | 2275 | 6 |
|  | 10. I am too tired to eat | 831 | 5 | 762 | 1 | 680 | 2 | 2273 | 8 |
|  | 11. I need help doing my usual activities | 834 | 2 | 762 | 1 | 680 | 2 | 2276 | 5 |
|  | 12. I am frustrated by being too tired to do the things I want to do | 834 | 2 | 762 | 1 | 680 | 2 | 2276 | 5 |
|  | 13. I have to limit my social activity because I am tired | 834 | 2 | 762 | 1 | 680 | 2 | 2276 | 5 |
|  | 4. I feel tired | 833 | 3 | 762 | 1 | 680 | 2 | 2275 | 6 |
|  | 5. I have trouble starting things because I am tired | 834 | 2 | 762 | 1 | 680 | 2 | 2276 | 5 |
|  | 6. I have trouble finishing things because I am tired | 833 | 3 | 762 | 1 | 680 | 2 | 2275 | 6 |
|  | 7. I have energy | 833 | 3 | 762 | 1 | 680 | 2 | 2275 | 6 |
|  | 8. I am able to do my usual activities | 834 | 2 | 762 | 1 | 680 | 2 | 2276 | 5 |
|  | 9. I need to sleep during the day | 834 | 2 | 762 | 1 | 680 | 2 | 2276 | 5 |
|  | 1. I feel fatigued | 834 | 2 | 762 | 1 | 680 | 2 | 2276 | 5 |
|  | 2. I feel weak all over | 834 | 2 | 762 | 1 | 680 | 2 | 2276 | 5 |
| **BLISS-52** | 3. I feel listless (“washed out”) | 836 | 29 | 810 | 75 | 688 | 177 | 2334 | 281 |
|  | 10. I am too tired to eat | 835 | 30 | 809 | 76 | 686 | 179 | 2330 | 285 |
|  | 11. I need help doing my usual activities | 842 | 23 | 814 | 71 | 687 | 178 | 2343 | 272 |
|  | 12. I am frustrated by being too tired to do the things I want to do | 843 | 22 | 812 | 73 | 687 | 178 | 2342 | 273 |
|  | 13. I have to limit my social activity because I am tired | 842 | 23 | 813 | 72 | 690 | 175 | 2345 | 270 |
|  | 4. I feel tired | 839 | 26 | 808 | 77 | 686 | 179 | 2333 | 282 |
|  | 5. I have trouble starting things because I am tired | 841 | 24 | 809 | 76 | 689 | 176 | 2339 | 276 |
|  | 6. I have trouble finishing things because I am tired | 841 | 24 | 812 | 73 | 688 | 177 | 2341 | 274 |
|  | 7. I have energy | 839 | 26 | 811 | 74 | 689 | 176 | 2339 | 276 |
|  | 8. I am able to do my usual activities | 840 | 25 | 810 | 75 | 689 | 176 | 2339 | 276 |
|  | 9. I need to sleep during the day | 841 | 24 | 810 | 75 | 687 | 178 | 2338 | 277 |
|  | 1. I feel fatigued | 839 | 26 | 814 | 71 | 690 | 175 | 2343 | 272 |
|  | 2. I feel weak all over | 843 | 22 | 812 | 73 | 689 | 176 | 2344 | 271 |
| **BLISS-76** | 3. I feel listless (“washed out”) | 810 | 9 | 737 | 91 | 617 | 203 | 2164 | 303 |
|  | 10. I am too tired to eat | 807 | 12 | 734 | 94 | 618 | 202 | 2159 | 308 |
|  | 11. I need help doing my usual activities | 809 | 10 | 734 | 94 | 618 | 202 | 2161 | 306 |
|  | 12. I am frustrated by being too tired to do the things I want to do | 809 | 10 | 735 | 93 | 619 | 201 | 2163 | 304 |
|  | 13. I have to limit my social activity because I am tired | 811 | 8 | 736 | 92 | 619 | 201 | 2166 | 301 |
|  | 4. I feel tired | 807 | 12 | 736 | 92 | 619 | 201 | 2162 | 305 |
|  | 5. I have trouble starting things because I am tired | 811 | 8 | 735 | 93 | 617 | 203 | 2163 | 304 |
|  | 6. I have trouble finishing things because I am tired | 811 | 8 | 735 | 93 | 618 | 202 | 2164 | 303 |
|  | 7. I have energy | 811 | 8 | 735 | 93 | 618 | 202 | 2164 | 303 |
|  | 8. I am able to do my usual activities | 809 | 10 | 734 | 94 | 617 | 203 | 2160 | 307 |
|  | 9. I need to sleep during the day | 811 | 8 | 733 | 95 | 619 | 201 | 2163 | 304 |
|  | 1. I feel fatigued | 810 | 9 | 737 | 91 | 619 | 201 | 2166 | 301 |
|  | 2. I feel weak all over | 811 | 8 | 736 | 92 | 619 | 201 | 2166 | 301 |

The FACIT-Fatigue was scored such that lower scores indicate greater fatigue and higher scores indicate less fatigue. FACIT-Fatigue total scores range from 0 to 52. To obtain the 0–52 score, each negatively worded item response is recoded so that 0 is a bad response and 4 is a good response. All responses are added with equal weight to obtain the total score. In cases where some answers may be missing, a total score is prorated from the score of the answered items provided that >50% of the items (i.e. at least 7 of 13) were answered. No imputation was conducted. N and n correspond to the number of patients in the study and the number of patients who dropped out of the study at each visit, respectively. As patients were required to complete FACIT-Fatigue, the numbers also indicate the number of patients who completed or did not complete the assessments.
