# Supplementary material for: Psychometric properties of FACIT-Fatigue in systemic lupus erythematosus: a pooled analysis of three phase 3 randomised, double-blind, parallel-group controlled studies (BLISS-SC, BLISS-52, BLISS-76)
Source: J Patient Rep Outcomes. 2021 Apr 8;5:33. doi: 10.1186/s41687-021-00298-x (PMC8032841; doi:10.1186/s41687-021-00298-x)
Supplement: Supplementary file 4 — Additional file 4: The confirmatory factor analysis method is described in the text before Supplementary Table S3. Consider adjusting the order and naming of the supplementary files. [file 41687_2021_298_MOESM4_ESM.docx]

# Confirmatory factor analysis (CFA)

A categorical CFA was conducted using data from the three BLISS studies of belimumab in patients with SLE to test whether the conceptual framework of the FACIT-Fatigue was verified in the sample of patients who participated in the three BeLimumab In Subjects with Systemic lupus erythematosus (BLISS) Phase 3 trials. Tests of the fit of the 13 items to a unidimensional, one-factor model, were conducted. Analyses were conducted in each of the three clinical trials separately using data from baseline and Week 24. The goodness-of-fit of the one-factor model was evaluated using the Comparative Fit Index (CFI), Tucker-Lewis Index (TLI), and the root mean square error of approximation (RMSEA). CFI and TLI are comparative fit indices, which quantify the amount of difference between the examined model and the independence model (i.e*.* a standard comparison model that asserts none of the components in the model are related), with higher scores indicating larger differences. It is recommended that these two indices be ≥0.9 as evidence of acceptable model fit.^1^ An RMSEA of 0 indicates perfect model fit.^1^

1. Hu, L., & Bentler, P. M. (1999). Cutoff criteria for fit indexes in covariance structure analysis: Conventional criteria versus new alternatives. *Struct Equ Modeling*, *6*, 1–55. 1.
