# Supplementary material for: Psychometric properties of FACIT-Fatigue in systemic lupus erythematosus: a pooled analysis of three phase 3 randomised, double-blind, parallel-group controlled studies (BLISS-SC, BLISS-52, BLISS-76)
Source: J Patient Rep Outcomes. 2021 Apr 8;5:33. doi: 10.1186/s41687-021-00298-x (PMC8032841; doi:10.1186/s41687-021-00298-x)
Supplement: Supplementary file 5 — Additional file 5: Supplementary Table S4. CFA tests of one-factor model, FACIT-Fatigue. [file 41687_2021_298_MOESM5_ESM.docx]

Supplementary Table S4. CFA tests of one-factor model, FACIT-Fatigue

|  |  | | **BLISS-SC** | | **BLISS-52** | | **BLISS-76** | |
| --- | --- | --- | --- | --- | --- | --- | --- | --- |
|  |  | | **Baseline** | **Week 24** | **Baseline** | **Week 24** | **Baseline** | **Week 24** |
| **Items** | **Content** | | n=827 | n=761 | n=812 | n=781 | n=790 | n=718 |
| 1 | Feel fatigued | | 0.85 | 0.90 | 0.80 | 0.85 | 0.89 | 0.93 |
| 2 | Feel weak all over | | 0.85 | 0.90 | 0.84 | 0.87 | 0.85 | 0.91 |
| 3 | Feel listless | | 0.85 | 0.91 | 0.84 | 0.85 | 0.86 | 0.90 |
| 4 | Feel tired | | 0.89 | 0.90 | 0.87 | 0.86 | 0.90 | 0.94 |
| 5 | Trouble starting things | | 0.92 | 0.94 | 0.91 | 0.94 | 0.93 | 0.96 |
| 6 | Trouble finishing things | | 0.92 | 0.93 | 0.87 | 0.91 | 0.90 | 0.94 |
| 7 | Have energy | | 0.60 | 0.68 | 0.62 | 0.65 | 0.71 | 0.77 |
| 8 | Able to do activities | | 0.60 | 0.64 | 0.61 | **0.59** | 0.65 | 0.76 |
| 9 | Need to sleep | | **0.57** | 0.60 | **0.40** | **0.47** | 0.62 | 0.65 |
| 10 | Too tired to eat | | 0.65 | 0.73 | **0.58** | 0.66 | 0.68 | 0.70 |
| 11 | Need help doing activities | | 0.75 | 0.79 | 0.67 | 0.70 | 0.74 | 0.80 |
| 12 | Frustrated being tired | | 0.85 | 0.89 | 0.82 | 0.83 | 0.84 | 0.89 |
| 13 | Limit social activities | | 0.85 | 0.88 | 0.84 | 0.84 | 0.86 | 0.89 |
| **Unidimensional model** | | | | | | | | |
| Model Fit |  | |  |  |  |  |  |  |
| CFI |  | | 0.96 | 0.96 | 0.95 | 0.96 | 0.97 | 0.98 |
| TLI |  | | 0.95 | 0.95 | 0.94 | 0.95 | 0.96 | 0.98 |
| RMSEA |  | | 0.15 | 0.17 | 0.15 | 0.14 | 0.14 | 0.14 |
| **Unidimensional model with correlated errors for 3 pairs of items: 5/6, 7/8, and 12/13** | | | | | | | | |
| Model Fit^a^ | |  |  |  |  |  |  |  |
| CFI | |  | 0.99 | 0.98 | 0.97 | 0.98 | 0.99 | 0.99 |
| TLI | |  | 0.98 | 0.98 | 0.96 | 0.98 | 0.99 | 0.99 |
| RMSEA | |  | 0.09 | 0.09 | 0.11 | 0.09 | 0.07 | 0.08 |

^a^Model Fit after freeing up correlations between pairs of items with high residual correlations.

*CFA*, confirmatory factor analysis; *CFI* Comparative Fit Index, *FACIT* Functional Assessment of Chronic Illness Therapy, *RMSEA* Root Mean Square Error of Approximation, *TLI* Tucker-Lewis Index.

Item-to-factor loadings were acceptable. Out of 78 item-to-factor loadings presented in **Supplementary Table S4**, only five (bolded) were <0.60. The fit statistics generally support a unidimensional model for FACIT-Fatigue items, confirming the conceptual framework of the FACIT-Fatigue in each of the BLISS trials in patients with SLE. Both CFI and TLI were >0.9 across trials and time points; however, the RMSEA was >0.10 in each of the initial CFAs. Further inspection of the CFA results revealed elevated residual correlations (r>0.10) between three pairs of items that contributed to the observed elevated RMSEA. The specific item pairs included: (1) items 7 (I have energy) and 8 (I am able to do my usual activities); (2) items 5 (I have trouble starting things because I am tired) and 6 (I have trouble finishing things because I am tired); and (3) items 12 (I am frustrated by being too tired to do the things I want to do) and 13 (I have to limit my social activity because I am tired). Based on the item content of each of these three pairs of items, the elevated residual correlates seem plausible. For the first item pair (7 and 8), both items are positively worded, whereas the remaining 11 items of FACIT are negatively worded. Thus, the positive wording of these two items likely accounts for a correlation between them not accounted for by the total scale. For the second pair of items (5 and 6), the wording is almost identical, resulting in residual correlation not accounted for by the total scale. Lastly, the third pair of items (12 and 13) both measure the extent to which tiredness limits the ability to do things or participate in social activities. The common theme between these two items is how being tired limits the individual’s ability to do things they want to do, which includes social activities. To address the elevated RMSEA observed in each of the initial CFAs conducted, the models were run again after freeing up the correlations between the three pairs of items. This alternative model specification relaxes the restriction that variation in these items is solely explained by fatigue (the latent variable) and random measurement error. The fit statistics for each of these models are presented at the bottom of **Supplementary Table S4**. As shown across all models, both CFI and TLI fit statistics increased. More importantly, by freeing up the correlations between the three pairs of items, the RMSEA was reduced to a level considered acceptable (0.10) in five of the six models.
