# Supplementary material for: Psychometric properties of FACIT-Fatigue in systemic lupus erythematosus: a pooled analysis of three phase 3 randomised, double-blind, parallel-group controlled studies (BLISS-SC, BLISS-52, BLISS-76)
Source: J Patient Rep Outcomes. 2021 Apr 8;5:33. doi: 10.1186/s41687-021-00298-x (PMC8032841; doi:10.1186/s41687-021-00298-x)
Supplement: Supplementary file 6 — Additional file 6: Supplementary Table S5. Model fit statistic for CFA tests of the two-factor model, FACIT-Fatigue. [file 41687_2021_298_MOESM6_ESM.docx]

Supplementary Table S5. Model fit statistic for CFA tests of the two-factor^a^ model, FACIT-Fatigue

|  | **BLISS-SC** | | **BLISS-52** | | **BLISS-76** | |
| --- | --- | --- | --- | --- | --- | --- |
|  | **Baseline** | **Week 24** | **Baseline** | **Week 24** | **Baseline** | **Week 24** |
| **Model fit** | n=827 | n=761 | n=812 | n=781 | n=790 | n=718 |
| CFI | 0.97 | 0.97 | 0.96 | 0.97 | 0.98 | 0.99 |
| TLI | 0.96 | 0.96 | 0.95 | 0.96 | 0.97 | 0.98 |
| RMSEA | 0.13 | 0.16 | 0.12 | 0.13 | 0.12 | 0.12 |
| Factor-Factor Correlation | 0.90 | 0.92 | 0.89 | 0.91 | 0.93 | 0.94 |

^a^Two-Factor Model consisted of assigning FACIT-Fatigue items to “Fatigue Experience” and “Fatigue Impact” based item content: Fatigue Experience (items: 1, 2, 3, 4, and 7); Fatigue Impact (items: 5, 6, 8, 9, 10, 11, 12, and 13).

*CFA,* confirmatory factor analysis*; CFI* Comparative Fit Index, *FACIT* Functional Assessment of Chronic Illness Therapy, *RMSEA* Root Mean Square Error of Approximation, *TLI* Tucker-Lewis Index.

To rule out that the elevated RMSEA in the initial models was not indicative of some multidimensionality among FACIT-Fatigue items, additional CFAs were conducted in each trial at baseline and Week 24. These additional CFAs tested a two-dimensional model, where items were assigned to measure either the experience of fatigue (items 1, 2, 3, 4, and 7) or fatigue impact. As shown in **Supplementary Table S5**, model fit statistics, in particular CFI and TLI, were comparable with the one-factor model fit statistics. On the other hand, the RMSEA statistics were all >0.10, a similar problem observed in the results of tests of the one-factor model. Lastly, as shown in **Supplementary Table S5**, the factor-to-factor correlations were very high, ranging from 0.89 to 0.94, confirming that the “fatigue experience” and “fatigue impact” measured by FACIT-Fatigue items are overlapping concepts. All combined, these results suggest that the two-factor model does not represent a better solution over the one-factor model for FACIT-Fatigue items.
